# Multi-view SoftPool attention convolutional networks for 3D model classification

**DOI:** 10.3389/fnbot.2022.1029968

**Published:** 2022-11-16

**Authors:** Wenju Wang, Xiaolin Wang, Gang Chen, Haoran Zhou

**Affiliations:** College of Communication and Art Design, University of Shanghai for Science and Technology, Shanghai, China

**Keywords:** 3D model classification, multi-view, attention, SoftPool, convolutional

## Abstract

**Introduction:**

Existing multi-view-based 3D model classification methods have the problems of insufficient view refinement feature extraction and poor generalization ability of the network model, which makes it difficult to further improve the classification accuracy. To this end, this paper proposes a multi-view SoftPool attention convolutional network for 3D model classification tasks.

**Methods:**

This method extracts multi-view features through ResNest and adaptive pooling modules, and the extracted features can better represent 3D models. Then, the results of the multi-view feature extraction processed using SoftPool are used as the Query for the self-attentive calculation, which enables the subsequent refinement extraction. We then input the attention scores calculated by Query and Key in the self-attention calculation into the mobile inverted bottleneck convolution, which effectively improves the generalization of the network model. Based on our proposed method, a compact 3D global descriptor is finally generated, achieving a high-accuracy 3D model classification performance.

**Results:**

Experimental results showed that our method achieves 96.96% OA and 95.68% AA on ModelNet40 and 98.57% OA and 98.42% AA on ModelNet10.

**Discussion:**

Compared with a multitude of popular methods, our algorithm model achieves the state-of-the-art classification accuracy.

## 1. Introduction

With the rapid development of 3D acquisition technology, various types of sensor devices (e.g., 3D scanners, LIDAR, and RGB-D cameras) can collect 3D data conveniently and quickly (Grenzdörffer et al., 2020). 3D data are abundant in geometry, shape, and scale information and simple in expression, so are well suited for 3D scene perception and understanding. 3D model-based classification is an important fundamental task in 3D visual perception tasks such as target segmentation, recognition and tracking, and matching. 3D model classification methods are currently extensively applied in the fields of robotics (Kästner et al., 2020), autonomous driving (Yu et al., 2021), 3D scene reconstruction (Pontes et al., 2017), augmented reality (Adikari et al., 2020), and medicine (Liu et al., 2020); hence, 3D model classification methods have become a research hotspot.

3D model classification methods can be divided into two fields: traditional and recent deep learning. Early 3D model classification tasks focused on hand-designed feature extraction followed by machine learning methods for classification (e.g., extreme learning machines and support vector learning). Lalonde et al. (2005) investigated the automatic data-driven scale selection problem using an approach driven by the Gaussian mixture model geometry. The method does not consider the relationship between neighbors, and the results are affected by noise, leading to poor classification accuracy. To solve these problems, Niemeyer et al. (2014) combined the contextual information and then embedded the random forest classifier into the conditional random field (CRE), which improved the classification accuracy to some extent. However, optimization is still essential in terms of feature extraction and the graph structure, as well as research on reducing the amount of data and the training time.

Traditional methods generally have several deficiencies, including limited manual feature extraction and low classification accuracy. Deep learning technology has achieved considerably good performance in computer vision, natural language processing, speech recognition, and other fields. In recent years, ModelNet (Wu et al., 2015), ShapeNet (Yang et al., 2021), ScanNet (Zou et al., 2021), and other publicly available datasets have also driven research in 3D model classification based on deep learning. 3D model classification methods based on deep learning can be divided into three categories based on the representation of the input data: voxel-based, point cloud-based, and multi-view-based.

### 1.1. Voxel-based methods

The voxel-based model method aims to voxelize the point cloud first, then employ a 3D convolutional neural network (CNN) to extract features, and finally complete the classification task. Maturana and Scherer (2015) proposed VoxelNet based on the idea of voxels, which is the voxelization of unstructured point cloud data into regular grid data for classification. The method corresponds each grid to a voxel, and the values in the grid cells are normalized and input to the convolutional layer in the network for feature extraction and classification. However, this method consumes a large amount of memory because of the large number of zero-valued voxels that appear in the process. Wu et al. (2015) proposed a convolutional deep belief network (3DShapeNet) for the classification of 3D models of different kinds and different poses. Both VoxNet and 3DShapeNet have the problems of prohibitive memory overhead in the computation and low accuracy of model classification. To reduce the memory consumption and running time, Riegler et al. (2017) proposed OctNet, a sparse 3D data representation method. The spatial stratification is represented as a series of unbalanced octree structures with pooled features stored on the leaf nodes in the octree. This method allows CNNs to handle high resolutions with reduced memory consumption, yet the problem of losing local geometric information has not been solved. Aiming to solve the problem, Wang et al. (2018) divided the whole space into voxels of different scales and employed the proposed multi-scale convolutional network (MSNet) to learn local features adaptively and fuse the local features to predict the class probability of the model. The network allows for improved classification accuracy and the ability to retain a large amount of information, but the training time of a voxelized grid can be exceedingly long. To reduce the time consumption, Le and Duan (2018) proposed the 3D convolutional grid PointGrid. It belongs to the regular embedded voxel grid, and the network can extract a large number of local features for 3D model classification.

In summary, the voxel-based method converts 3D point clouds into voxel meshes, solving the problem of unstructured 3D point clouds. However, as the voxelization requires the input voxel format to be regular for a convolution operation, a large amount of information is lost when the voxel resolution is low, which causes the problem of low classification accuracy. Moreover, it has the problem of high computational cost when the resolution is high.

### 1.2. Point cloud-based methods

The point cloud-based method aims to directly classify point cloud data obtained by 3D scanners, LIDAR, and RGB-D cameras using the corresponding approaches. Qi et al. (2017a) considered the direct processing of point cloud data and proposed the PointNet network, which transforms the input point cloud through the T-Net matrix and applies the multilayer perceptron (MLP) to learn the features of the points and aggregate them into global features. Their experiment and analysis showed that PointNet made a great breakthrough in point cloud classification and segmentation, but it could not capture local information and had poor generalization ability. PointNet++ (Qi et al., 2017b) was proposed based on the shortcomings of PointNet in recognizing fine-grained patterns. By introducing a hierarchical neural network and metric spatial distance, the context ratio can be increased, and thus the network can better learn local features. The introduced ensemble learning layer can adaptively combine multiple scale features for classification. Nevertheless, this method lacks some structural information between points. Ma et al. (2018) proposed the 3DMAX-Net architecture influenced by the contextual information mechanism. This network can obtain the contextual features in 3D point cloud space through the introduced multi-scale feature learning block, while the features learned by the network are aggregated through a local-global feature aggregation block. Qiu et al. (2021) proposed a density resolution network by introducing an adaptive extended point algorithm; an error minimization module in the network is utilized to extract multi-resolution information, and local features are fused to achieve the point cloud classification task. The classification accuracy of the model was shown to be higher than that in the PointNet network. Additionally, both 3DMAX-Net network and density-resolution network are not applicable to large-scale point clouds; they are also especially insufficient in the case of many object classes.

To address the problem that most networks cannot adapt to large-scale point clouds, Hu et al. (2020) proposed RandLA-Net, which is based on a complex sampling technique that devises random point sampling to reduce computation and memory, while the introduced local feature aggregation blocks retain important information among neighbors. RandLA-Net can directly handle large-scale point clouds, and using a lightweight network can improve classification accuracy while greatly reducing the computational memory and time overhead. However, because the RandLA-Net network chooses random sampling, there is a loss of useful information. Liang et al. (2019) proposed a deep graph CNN for local geometric feature extraction, which obtains a large amount of useful information and has a smaller memory consumption compared to previous graph convolution methods. Zhang et al. (2020) proposed an omnidirectional graph neural network for further improving the performance of the network and reducing the complexity of the model. The method proposes LKPO-GNN for obtaining local and global spatial information, learning the local topology of the point cloud using the omnidirectional local KNNs pattern, and aggregating the local information spatial structure to obtain the global map using GNN. In contrast, the KNN pattern still has defects in neighborhood search. Feng et al. (2020) considered the lack of performance in neighborhood search and constructed local graphs based on searching neighborhood points in multiple directions while assigning attention coefficients to each edge of the graph and aggregating centroid features as a weighted sum of its neighboring points to obtain local features. Moreover, the point-by-point spatial attention module is used to generate the interdependency matrix of points so that local features and contextual information can be obtained simultaneously. The performance of this method is enhanced in point cloud classification and segmentation. Wen et al. (2020) proposed a novel deep learning network of Point2SpatialCapsule based on aggregating local features and spatial relationships of point clouds. This network consists of two modules, geometric feature aggregation, and spatial relationship aggregation, which are capable of aggregating local features to clustering centers and aggregating their spatial relationships in the feature space using spatially aware capsules. This method has greatly elevated the accuracy of tasks (e.g., point cloud classification retrieval).

However, owing to the disorderly and unstructured nature of 3D point clouds, as well as the fact that scanned models in real scenes can be obscured and result in partial data loss and complex scenes, direct methods of processing point clouds are often more complex and take longer to train.

### 1.3. Multi-view-based methods

The multi-view-based method aims to project the 3D model from multiple virtual cameras into the 2D plane and then perform convolutional feature extraction and fusion on the obtained multi-views to accomplish the task of 3D model classification. The earliest rendering of 3D point clouds into multi-views and applying them to model classification is the MVCNN network proposed by Su et al. (2015). The classification accuracy and performance of MVCNN represent a remarkable breakthrough in point cloud classification, but because of the maximum pooling, keeping only the largest elements in these views can lead to a large amount of information loss. To reduce the loss of effective information, Wang et al. proposed RCPCNN (Wang C. et al., 2019) to perform dominant set clustering from the views of the same cluster. RCPCNN is updated iteratively in the pooling layer in a round-robin fashion. This method improves the classification performance but ignores the relationships among views. Feng et al. (2018) introduced a hierarchical view-group-shape framework, called GVCNN, which is based on MVCNN to better utilize the connection between multiple views. It can find more discriminative features among views and offers a significant improvement in classification accuracy. Yet, this method relies too much on the choice of the viewpoint angle and is not applicable to the case of a small number of views. Yu et al. (2018) proposed MHBN using the relationship between the polynomial kernel and bilinear pool and considered that local complementary information exists among different views. Bilinear pooling aggregates local features to measure similar pairs of related patch pairs and coordinates the merging of bilinear features to generate a more discriminative 3D object representation. MHBN offers an improvement in classification accuracy and storage efficiency, and also effectively suppresses irrelevant matching pairs. Ma et al. (2019) combined CNNs with long short-term memory (LSTM) based on the sequential nature among views and used LSTM and sequential voting layers to aggregate multi-view features into shape descriptors for object recognition.

Han et al. (2019b) proposed the SeqViews2SeqLabels network considering the spatial relationship of views. It is composed of an encoder for aggregating sequence views and a decoder for global feature prediction sequence labels. An attention mechanism is incorporated in this decoder, and specific views are assigned more weights to improve the discriminative ability. Moreover, better classification accuracy is obtained. For this reason, they further proposed the 3D2SeqViews network (Han et al., 2019a), which has more novel hierarchical attention to efficiently aggregate the content information of views and spatially related information between views. It affords great progress in global feature aggregation. However, CNN and LSTM combined with SeqViews2SeqLabels networks can only aggregate ordered views, not unordered views. Based on this problem, Yang and Wang (2019) proposed a relational network from the perspective of relationships among different view regions and views. The training methods effectively connect the corresponding regions through the self-attention module, combining the inter-view relationships to highlight the salient information more, which can enhance the information of single-view images. In contrast, there are still shortcomings in the selection of relationships among views, and selecting views that do not overlap and just complement each other still needs to be studied further. To improve the generalization ability and performance of the model, Sun et al. (2021) proposed a dynamically routed CNN. The method is based on a dynamic routing algorithm for adaptive selection of features for transformation, which does not ignore the inconspicuous information in the pooling layer and effectively fuses the features of all views. Wei et al. (2020) proposed view-GCN from the perspective of graph convolution. It is a hierarchical network based on view-graph representation, which is a viewgraph constructed by using multiple views as graph nodes and sampling representative views by the introduced view selection mechanism. The local and non-local convolution of this network performs feature transformation, which can obtain 3D object descriptors with different levels of feature combinations. Yet, this network is less flexible and scalable for shallow GCNs, and cannot pass the labels with little training data to the whole graph structure. On this basis, Liu et al. (2021) proposed a hierarchical multi-view context modeling approach, which consists of four main components: view-level context learning, the multi-view grouping module, the primitive group level, and the group fusion module. The method can fuse group-by-group contextual features into compact 3D object descriptors for object classification according to their importance.

So far, the view-based approach has achieved the best results on 3D model classification tasks. Compared to the direct point cloud and voxel processing approach, it can capture the features of the view more easily and learn the view features to synthesize true global feature descriptors with the help of a proven CNN. However, the method still has shortcomings in feature extraction, because the traditional pooled downsampling method cannot treat each view equally and only retains the information considered important. This leads to the problem of the insufficient extraction of view refinement feature information and the loss of a large amount of view feature information. However, different convolutional models learn different classification rules through a given dataset, so the classification accuracy predicted by the network model for unknown datasets varies greatly. Therefore, different convolutional models do not have the same degree of generalization. Both insufficient extractions of view refinement feature information and weak model generalization affect the further improvement of 3D model classification accuracy. Based on the above analysis, we propose a multi-view SoftPool attention convolutional network framework (MVMSAN) for 3D model classification tasks. Compared with traditional methods, our method employs a SoftPool attention convolution framework that can extract refined view feature information, effectively solving the problem of feature information loss and insufficient detail feature extraction during downsampling while enhancing the generalization ability of the model. Thus, the framework improves the accuracy of 3D model classification.

This study made the following contributions:

(1) We propose the MVMSAN network framework. It employs ResNest with the adaptive pooling method, SoftPool attention method, and self-attention convolution method to generate discriminative global descriptors for 3D model classification. Compared with a multitude of popular methods, our network framework achieves the state-of-the-art classification accuracy.

(2) ResNest with the adaptive pooling method removes the last fully connected layer and adds an adaptive pooling layer. This method can be applied to the extraction of view feature information, which focuses more on the feature information among view channels, reinforces the representation of feature maps, and better obtains real 3D features from 2D views.

(3) The SoftPool attention method can obtain finer view feature information, emphasize the importance of detailed features, and obtain more distinguishing features with model categories, because SoftPool uses the processed view feature value as the Query value of the self-attention. The self-attention-based convolution method can also improve the generalization ability of the model and focus on the learning ability of the algorithmic framework to increase the accuracy of 3D model classification, because Mobile inverted Bottleneck Convolution (MBConv) is used to process the Query and Key of self-attention.

(4) Our extensive experiments on the ModelNet40 and ModelNet10 datasets demonstrate the effectiveness of the proposed method. The experimental results show that, compared with existing state-of-the-art classification methods, the overall classification accuracy of our method on the two datasets reaches 96.96 and 98.57%, respectively.

## 2. Methods

**Figure 1:**
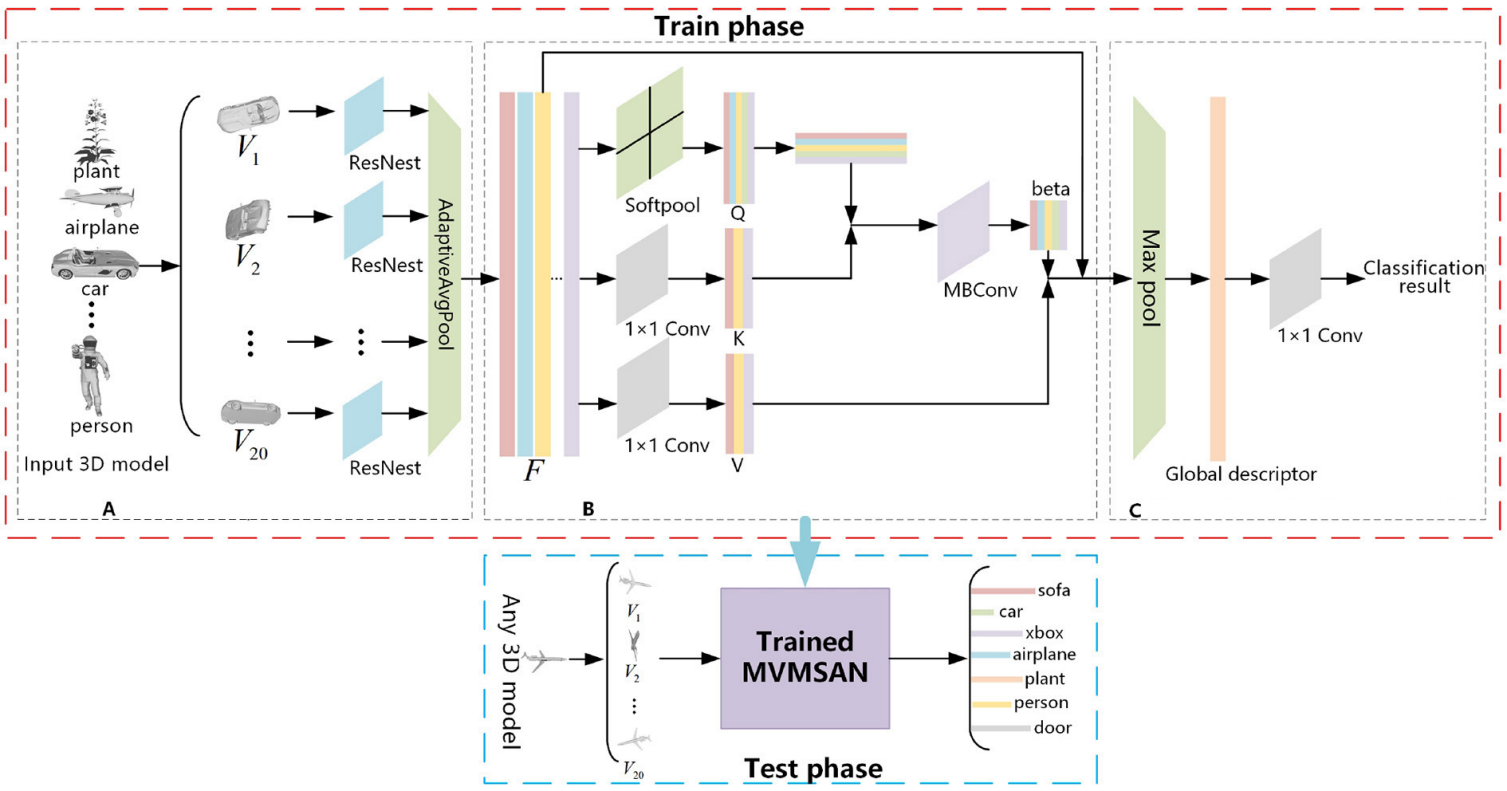
Multi-view SoftPool attention convolution (MVMSAN) network framework. **(A)** Multi-view acquisition module. **(B)** Multi-view refined feature extraction module. **(C)** Feature fusion classification module.

The framework diagram Multi-view SoftPool Attention Convolution (MVMSAN) proposed by us is divided into three modules (Figure 1): the 3D model multi-view acquisition module, multi-view refinement feature extraction module, and feature fusion classification module. The multi-view acquisition module presents the 3D model in multiple views. The multi-view refinement feature extraction module employs ResNest with an adaptive pooling method to extract the feature information of the view. Then it uses our proposed SoftPool attention convolution method for view feature refinement extraction, which enables the subsequent fusion to generate more compact global descriptors. The feature fusion classification module aggregates refined features through pooling layers to generate global representation and completes 3D model classification by 1 × 1 convolution. The MVMSAN network framework will obtain a trained classification network model in the training phase, which uses datasets including ModelNet40 and ModelNet10 as training data. Any 3D mesh model can be input into the MVMSAN classification model trained for classification prediction in the testing phase.

### 2.1. 3D model multi-view acquisition

Our input is a mesh, point cloud representation of the 3D model. Then, a set of images from different angles *V* = {*v*_1_, …*v*_*i*_…, *v*_20_} are used instead of the virtual 3D model, where *V*_*i*_ denotes the 2D images generated from 1 to 20 different viewpoint angles for any 3D model. The process applies the viewpoint selection method proposed by Kanezaki et al. (2018), which involves placing the 3D model at the center of the ortho dodecahedron and 20 virtual cameras on 20 vertices of the ortho dodecahedron. The dodecahedron is chosen because it has the highest number of vertices among the ortho polyhedra, and all viewpoints are evenly distributed in the 3D space where the 3D model is located.

### 2.2. Multi-view refinement local feature extraction

#### 2.2.1. Extraction of view features based on ResNest with the adaptive pooling method

For the 20 views *V* = {*v*_1_, …*v*_*i*_…, *v*_20_} obtained from the 3D model rendering, we use ResNest (Zhang et al., 2022) to extract the view features. ResNest is based on ResNet with the addition of split-attention blocks, which can exploit the interrelationship among view channels. Thus, it increases the perceptual field of feature extraction, strengthens the representation of feature maps, and reduces information loss. The view feature information extracted by ResNest is denoted as {*m*_1_, …*m*_*i*_…, *m*_20_}. See Equation (1):


(1) $$\{\begin{array}{r} m_{1}=ResNest\left(v_{1}\right)\\ \vdots \\ m_{i}=ResNest\left(v_{i}\right)\\ \vdots \\ m_{20}=ResNest\left(v_{20}\right) \end{array}$$

where {*m*_1_, …*m*_*i*_…, *m*_20_} denotes the 20 extracted view features.

All the view features are stitched together to obtain the following Equation:


(2) $$\begin{array}{l} \;M=\overset{i=20}{\underset{i=1}{\sum }}ResNest\left(v_{i}\right) \end{array}$$

To satisfy the data input requirements for the subsequent SoftPool attention convolution processing (Section 2.2.2), we propose a combination of ResNest and adaptive pooling for view feature extraction. In this method, ResNest removes the final fully-connected layer and adds an AdaptiveAvgPool2d process. This is because adaptive pooling can obtain the output of a specified size based on an input, and the number of features in the input and output does not change. Therefore, the output of ResNest after adaptive pooling ensures that the view feature information extracted by the network remains unchanged and also satisfies the input requirements for the subsequent SoftPool attention convolution.

The view features extracted by ResNest are processed by the adaptive pooling layer to obtain *F*, as shown in Equation (3):


(3) $$\begin{array}{l} \;F=AAP\left(M\right) \end{array}$$

#### 2.2.2. Refined feature extraction based on SoftPool attention convolution

**Figure 2:**
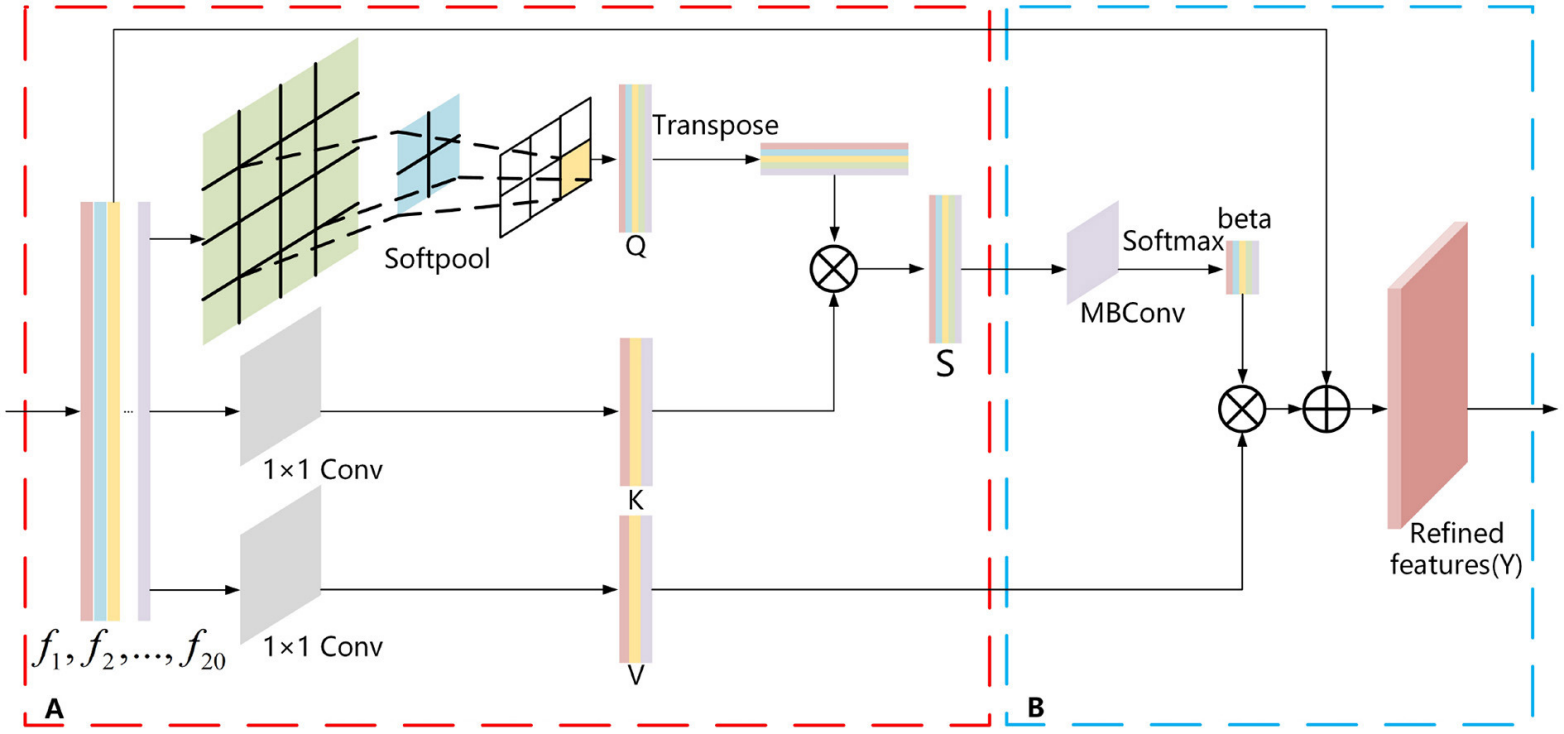
SoftPool attention convolution method. **(A)** Refinement feature extraction based on SoftPool. **(B)** Model generalized enhancement based on self-attention convolution.

There is also some unnecessary information in the view features (*F*) extracted using ResNest with the adaptive pooling method. This information is redundant for aggregation into a global descriptor. For this purpose, we propose a SoftPool attention convolution method to accomplish refined feature extraction. This method mainly relies on the self-attention mechanism (Zhang et al., 2019). As self-attention can process the entire input view feature information globally, its strong global perception capability enables global feature extraction of view features. However, it is deficient in the refinement extraction of local features of the view. Moreover, it lacks the inductive bias property, so it has poor generalization. Also, our proposed SoftPool attention convolution method solves these problems and can achieve fine-grained extraction of view features. It contains the following two modules: Refinement feature extraction based on the SoftPool self-attention method; and Model generalization enhancement based on self-attention convolution (Figure 2).

#### 2.2.3. Refined feature extraction based on softPool self-attention method

The pooling layer used in most neural networks is either max pooling or average pooling. Max pooling selects only the max activation values in the region, resulting in a large amount of information loss. In contrast, average pooling averages all activation values, which reduces the overall region characteristics. Therefore, it is not appropriate to choose either max pooling or average pooling for view feature extraction. The SoftPool method (Stergiou et al., 2021) first selects the activation graph, divides the individual activation values in the activation graph by the sum of the natural exponents of all activation values to obtain the corresponding weight values, multiplies all the weights by the corresponding activation values, and sums them to obtain the output. This makes all activation values of the feature map act on the final output, which is the greatest difference between SoftPool and max and average pooling. To this end, this paper proposes the SoftPool self-attention method, which makes full use of the strong global perception capability of self-attention and preserves the detailed information of multi-view features by using SoftPool. The self-attention mechanism obtains the corresponding *V*-value after calculating the similarity between *Q* and *K* vectors, and then the *V*-value is weighted and summed to obtain the value of the self-attention method. In this method, SoftPool uses the processed view feature *F*-value as the *Q* value of self-attention, which can refine the multi-view feature downsampling process and retain more multi-view feature detail information to achieve refined feature extraction (Figure 2A). It effectively overcomes the shortage of the self-attention mechanism in viewing the local feature refinement extraction and helps to generate ultimate global descriptors with discriminative ability.

The process is divided into two steps:

(1) For the *F* = {*f*_1_, …*f*_*i*_…, *f*_20_} view features extracted by ResNest with the adaptive pooling method, *f*_*i*_ denotes the feature of the i-th view. We take the view feature (*F*) as input and generate a feature map (*Q*) by SoftPool (Stergiou et al., 2021) processing. Two 1 × 1 convolutions are also used to generate the feature maps *K* and *V*. See Equations (4), (5), and (6):


(4) $$\begin{array}{l} Q=SoftPool\left(F\right) \end{array}$$

(5) $$\begin{array}{l} K=Conv_{1\times 1}\left(F\right) \end{array}$$

(6) $$\begin{array}{l} V=Conv_{1\times 1}\left(F\right) \end{array}$$

where *F* denotes the feature vector of size m × n, *Conv*_1×1_ is a 1 × 1 convolution kernel, *K* and *V* are the feature vectors obtained by the 1 × 1 convolution operation, and *Q* is the feature vector obtained by the output of the SoftPool operation.

(2) The vector *S* is obtained by multiplying the vector *K* with the transpose vector *Q*^*T*^, as shown in Equation (7):


(7) $$\begin{array}{l} \;S=K\times Q^{T} \end{array}$$

where *T* is the transpose operation, × is the product operation between two vectors, and *S* denotes the matrix vector of the multiplication of *K* and *Q*^*T*^.

#### 2.2.4. Model generalization enhancement based on self-attention convolution

The self-attention mechanism has weak generalization owing to the lack of inductive bias (Dai et al., 2021). In contrast, convolution has good generalization ability owing to its convolution kernel, which is static and possesses translational invariance. To this end, we introduce the mobile inverted bottleneck convolution (MBConv) (Sandler et al., 2018), which is currently the most advanced convolution, in the self-attention mechanism to enhance the generalization (Figure 2B). The main principle of MBConv is that the input features are first up-dimensioned using 1 × 1 convolution, and then the information between their length and width is extracted by depth-separable convolution. The dimensionalized input feature information is downscaled by point convolution to obtain information across channels. A linear activation function is adopted in the dimensionality reduction process to prevent information loss. To prevent network degradation, a reversal residual block is added at the end to sum the reduced-dimensional features with the input features, which significantly improves the generalization performance of the model.

The process is divided into two steps.

(1) Input the vector *S* into *MBConv* (Sandler et al., 2018) and use the *SoftMax* function for scaling and normalization to obtain the attention weight values, as follows:


(8) $$\begin{array}{l} \;beta=Softmax\left(\frac{MBConv\left(S\right)}{\sqrt{d_{k}}}\right) \end{array}$$

(2) Take this attention weight value and multiply it with the *V* vector to obtain the result of the self-attention calculation *O* :


(9) $$\begin{array}{l} \;O=beta\times V \end{array}$$

where *beta* denotes the attention weights obtained by passing the *S* matrix through the *SoftMax* function, *SoftMax* is the activation function, and $\sqrt{d_{k}}$ is used to prevent the *S* value from being too large when the dimensionality is large.

We combine ResNest with the multi-view features (*F*) obtained by the adaptive pooling method with the result of the self-attention calculation (*O*) to finally obtain the refined features (*Y*) extracted by the SoftPool attention convolution method:


(10) $$\begin{array}{l} \;Y=F+gamma*O \end{array}$$

where *gamma* is the parameter, and *Y* denotes the refined features.

### 2.3. Feature fusion classification

**Figure 3:**
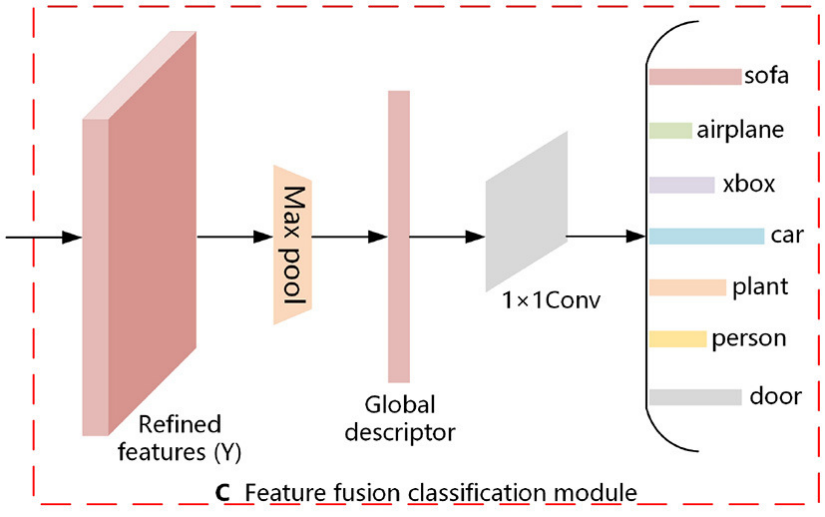
Feature fusion classification.

In this section, we describe the multi-view feature fusion classification module. It is shown in Figure 3. For the refined features (*Y*) obtained from the above equation, *Maxpooling* is utilized to aggregate the features and thus generate a compact global descriptor (*Global*), as shown in Equation (11). The 1 × 1 convolution allows the number of channels to be reduced by controlling the number of convolution kernels, and it does not limit the size of the input features. Therefore, we input the generated global descriptor (*Global*) to the 1 × 1 convolution to obtain the result of the 3D model classification, as shown in Equation (12).


(11) $$\begin{array}{l} \;Global=Max\left(Y\right) \end{array}$$

(12) $$\begin{array}{l} Z=Conv_{1\times 1}\left(Global\right) \end{array}$$

where *Z* denotes the result of the determination of N classes of objects, *Max* denotes the pooling aggregation operation, *Global* denotes the resulting global descriptor, and *Conv*_1×1_ denotes the convolution operation with a 1 × 1 convolution kernel.

## 3. Experiment

### 3.1. Datasets

To evaluate the performance of our proposed MVMSAN network, we conducted extensive classification comparison experiments using the ModelNet40 and ModelNet10 datasets. ModelNet40 includes 3D CAD models in 40 common grid forms, including 9,843 training models and 2,468 testing models. ModelNet10 contains 10 categories of 3D CAD models, with 3,991 training models and 908 testing models.Since the number of models varies across categories, we chose the overall accuracy OA (Uy et al., 2019; Equation 13) for each sample and the average accuracy AA (Zhai et al., 2020) (Equation 14) for each category as metrics to evaluate the classification performance. It is noteworthy that OA is the ratio of the number of correctly classified samples to the total number of samples, and AA is the average of the ratio of the number of correct predictions to the total number of predictions for each category. See Equations (13) and (14) for details.


(13) $$\begin{array}{l} \;OA=\frac{1}{N}\sum_{i=1}^{c}x_{ii} \end{array}$$

(14) $$\begin{array}{l} AA=\frac{sum\left(recall\right)}{C} \end{array}$$

where *N* is the total number of samples, *x*_*ii*_ is the number of correct classifications, and *C* denotes the category of the dataset, and recall denotes the ratio of predictions to samples.

### 3.2. Experimental setup and analysis

We conducted our experiments using a computer with Windows 10, Inter 8700K CPU, 64 GB RAM, and the RTX2080 graphics card. In all experiments, our environment was set to PyTorch 1.2 (Paszke et al., 2017) and Cuda 10.0. The experiment was divided into two training phases. The first phase classified only a single view to enable fine-tuning of the model while removing the SoftPool attention convolution module. The second stage added SoftPool attention convolutional blocks to train all views of the 3D model, which was used to train the whole classification framework. We only performed test experiments in the second stage and set 20 epochs. We optimized the entire network architecture using the Adam (Zhang, 2018) optimizer. The initial learning rate and L2 regularization weight decay parameters were set to 0.0001 and 0.001, respectively, to accelerate model convergence and reduce model overfitting.

### 3.3. Impact of CNN on classification performance

A pretrained CNN is used as a backbone model to improve the performance of various tasks, e.g., classification and segmentation. To extract view feature information more quickly and effectively, we connected the SoftPool attention convolution module to the encoders, such as ResNet18 (He et al., 2016), Densenet121 (Huang et al., 2017), ResNest50d, ResNest26d, and ResNest14d, in the ModelNet40 and ModelNet10 datasets. The experimental results are shown in Table 1. On the ModelNet40, the whole network had the shortest training time when using ResNet18, while the network deepened and the training time prolonged when using DenseNet121 and ResNest50d. In particular, the training process of the ResNest50d network model took 809 min (312 min more than ResNest14d). Employing ResNest14d as the backbone model, the OA and AA metrics of the MVMSAN network reached 96.96% and 95.68%, respectively, achieving the best classification performance. Hence, we chose ResNest14d as the backbone model for extracting multi-view features.

**Table 1:** Effects of different backbone models on classification performance.

**Network**		**ModelNet40**			**ModelNet10**		
		**Tim(min)**	**OA(%)**	**AA(%)**	**Tim(min)**	**OA(%)**	**AA(%)**
Resnet18		**366**	96.31	94.43	**147**	98.45	98.22
Densenet121		748	96.59	94.81	290	98.23	97.98
ResNest50d		809	96.31	94.22	327	98.24	98.07
ResNest26d		599	96.72	95.33	239	**98.67**	**98.45**
ResNest14d		497	**96.96**	**95.68**	200	98.57	98.42

The bold values represent the best performance.

### 3.4. The effect of different number of views on classification performance

**Figure 4:**
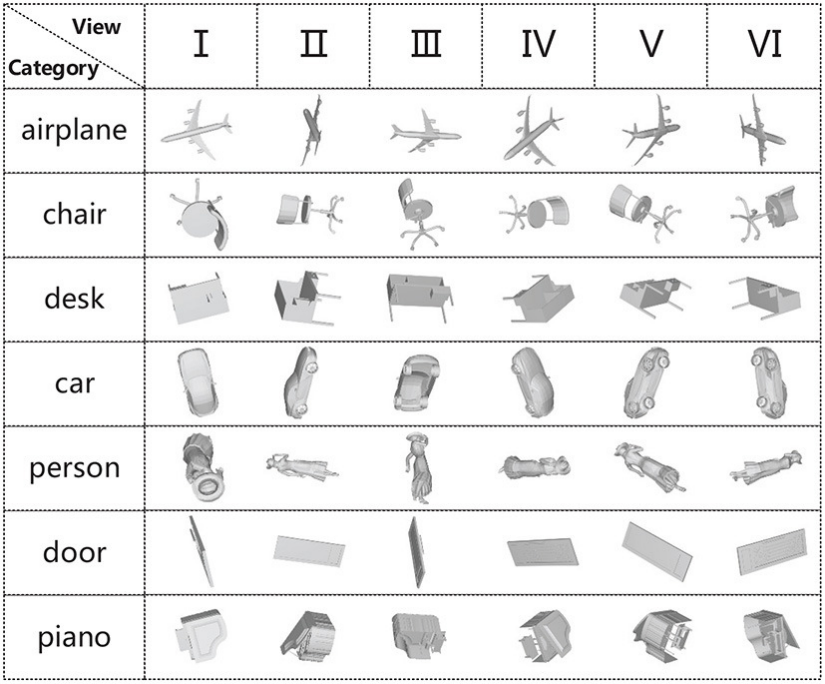
Six views of different models.

To more intuitively observe the view feature information in different angles, we selected 2D views of seven different categories of 3D models for display. As shown in Figure 4, the view V in the piano category ignores the key feature information of the keys; therefore, if a single view is used for experiments, the loss of feature information will affect the classification accuracy. Multiple views can fuse the feature information of different views to make up for the loss of single view feature information. To further investigate the effect of the number of views on the model classification performance, we randomly selected 3, 6, and 12 views from the 20 views obtained from 20 viewpoint angles for each 3D model in experiments. At the same time, the classification performance of MVMSAN was also compared with other advanced methods [such as MVCNN (Su et al., 2015), RCPCNN (Wang C. et al., 2019), 3D2SeqViews (Han et al., 2019a), VERAM (Chen et al., 2019), MHBN (Yu et al., 2018), and RN (Yang and Wang, 2019)] under 3, 6, and 12 number of views. The experimental results are shown in Table 2.

**Table 2:** The effect of the number of views on classification performance.

**Methods**	**ModelNet40**		
	**3 views**	**6 views**	**12 views**
MVCNN	91.33	92.01	91.49
RCPNN	92.10	92.22	92.18
3D2SeqViews	92.10	93.07	93.40
VERAM	92.40	93.30	93.70
MHBN	93.78	94.12	93.42
RN	93.50	94.10	94.30
MVMSAN(Ours)	**96.35**	**96.84**	**96.80**

The bold values represent the best performance.

On the ModelNet40 dataset, MVMSAN network outperformed other methods (such as MVCNN, RCPCNN, 3D2SeqViews, VERAM, MHBN, and RN). Compared with the RN network, our network improved OA by 3.0, 2.8, and 2.6% in each view configuration. In comparison with the classic MVCNN network, it improved by 5.2, 5.0, and 5.5%, respectively. From Table 2, we can see that the classification accuracy did not increase with the number of views; for example, our method achieved the best experimental results in six views. Meanwhile, it can be seen from the Table 2 that OA of our MVMSAN model can still reached 96.35, 96.84, and 96.80% in 3, 6, and 12 views. This experiment shows that our network has high robustness.

The high robustness achieved by the MVMSAN model is mainly attributed to our proposed SoftPool attention convolution method. SoftPool uses the processed view feature value as the Query value of the self-attended to obtain refined view feature information. Under any number of 1–20 views, these fine-grained view features can hold salient features related to model categories. Subsequent Mobile inverted bottleneck convolution (MBConv) can process the Query and Key of self-attentive, which significantly improve the generalization performance of MVMSAN model. The learning ability for our model also becomes stronger, so that it can achieve high classification accuracy with any number of 1–20 views.

### 3.5. Ablation experiments

We supplement a set of ablation experiments to demonstrate the generalization performance of SoftPool attentional convolution method proposed by us (see Tables 3, 4). The experimental results on the ModelNet40 dataset show that our proposed SoftPool attentional convolution method achieved the best classification performance on ModelNet40 (96.96% for OA and 95.68% for AA). The OA and AA obtained by applying only the output of SoftPool as the Query vector of attention were 96.11 and 94.47%, respectively, which were lower than those of the SoftPool attention convolution method. This is because the network model at this point is less generalizable, i.e., the classification ability learned by this network from the training set performs poorly. Adopting only MBConv to process the computational results of Query and Key of attention led to an insufficient feature extraction capability of the network. The loss of this feature information further reduced the classification accuracy (96.40 and 94.62% for OA and AA, respectively). We also obtained consistent experimental results on the ModelNet10 dataset (see Table 4).

**Table 3:** Ablation study (ModelNet40).

**ATT**	**Soft**	**MBConv**	**OA(%)**	**AA(%)**
✓			96.43	94.70
✓	✓		96.11	94.47
✓		✓	96.40	94.62
✓	✓	✓	**96.96**	**95.68**

ATT represents attention calculation, Soft represents the SoftPool method, and MBConv represents the mobile inverted bottleneck convolution. The bold values represent the best performance.

**Table 4:** Ablation study (ModelNet10).

**ATT**	**Soft**	**MBConv**	**OA(%)**	**AA(%)**
✓			98.34	98.20
✓	✓		98.23	97.98
✓		✓	98.24	97.99
✓	✓	✓	**98.57**	**98.42**

ATT represents attention calculation, Soft represents the SoftPool method, and MBConv represents the mobile inverted bottleneck convolution. The bold values represent the best performance.

It further proves that the best performance of the entire model can be achieved with the output result of SoftPool as the Query value of attention and MBConv to process the computational results of Query and Key of attention. It is worth noting that our algorithm can achieve 96.96% on OA and 95.68% on AA. The result is closely related to the refined feature extraction of SoftPool self-attention method and the model generalization enhancement of self-attention convolution method. The above two factors are indispensable.

We also employed a 1 × 1 convolution alternative to the fully connected layer that the network ends up using for classification. As shown in Table 5, the OA and AA using 1 × 1 convolution reached 96.96 and 95.68%, respectively, which is 0.17 and 0.48% improvement compared with fully connected layers. By using 1 × 1 convolution with fewer parameters, the training time in the same environment was also reduced by 27 min.

**Table 5:** Comparison of the effect of 1 × 1 convolution on classification performance.

**Network**	**OA(%)**	**AA(%)**	**Time(min)**
FC	96.79	95.20	524
1 × 1Conv	**96.96**	**95.68**	**497**

The bold values represent the best performance.

### 3.6. Confusion matrix visualization

**Figure 5:**
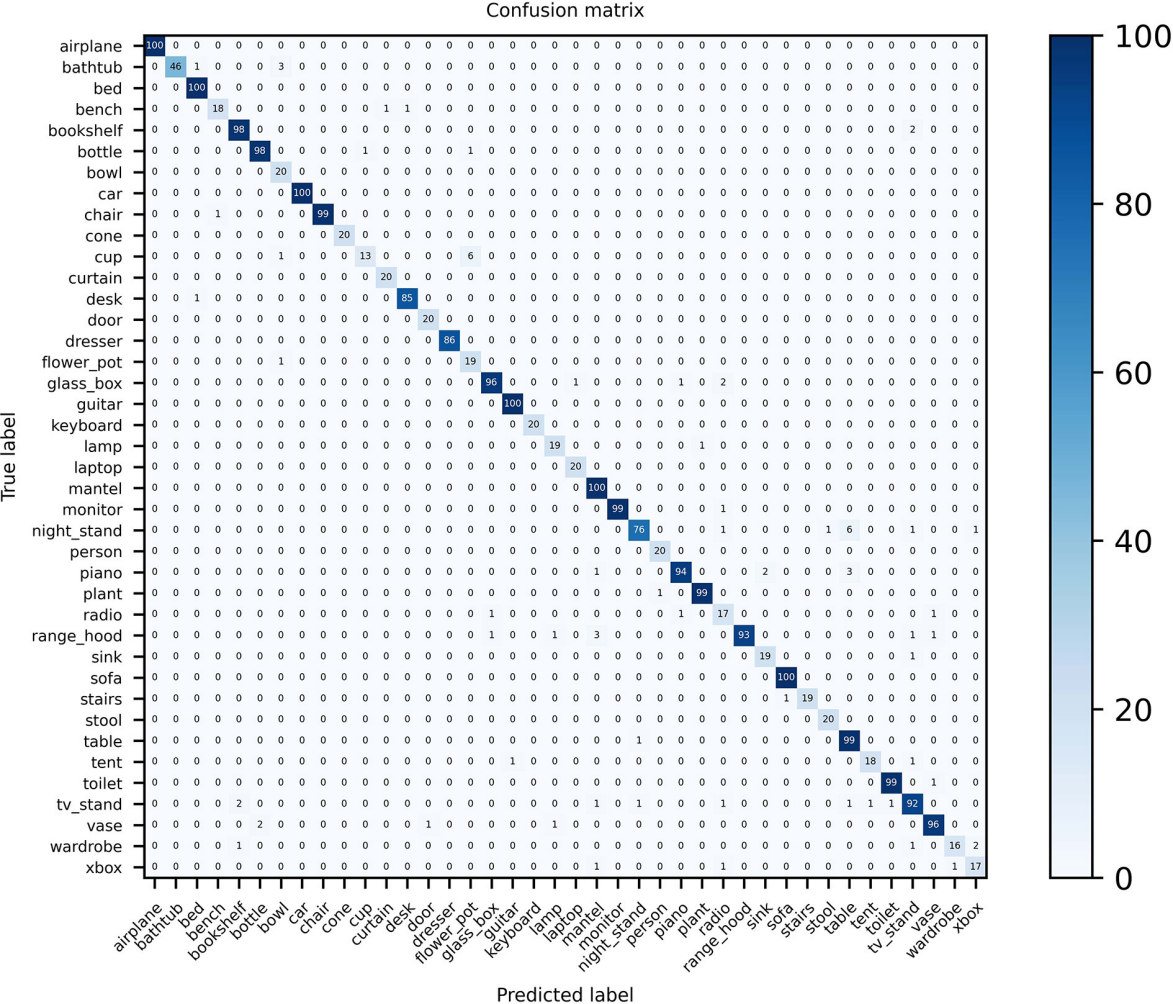
Confusion matrix visualization of MVMSAN on ModelNet40.

Confusion matrix visualization can intuitively demonstrate the advanced performance of the MVMSAN method on the 3D model classification task. Especially in the case that some view features have high similarity, our method still has high classification prediction performance. We plot the confusion matrix on the ModelNet40 and ModelNet10 datasets. On ModelNet40, it can be seen from Figure 5 that MVMSAN achieved 100% classification accuracy on categories such as airplane, bed, sofa, and guitar. In some harder categories, such as night stand, table, and xbox, some views have high similarity. In this case, our MVMSAN model can also classify correctly. It can be seen from Figure 5 that 76 samples are correctly classified among the 86 the night stand models.

**Figure 6:**
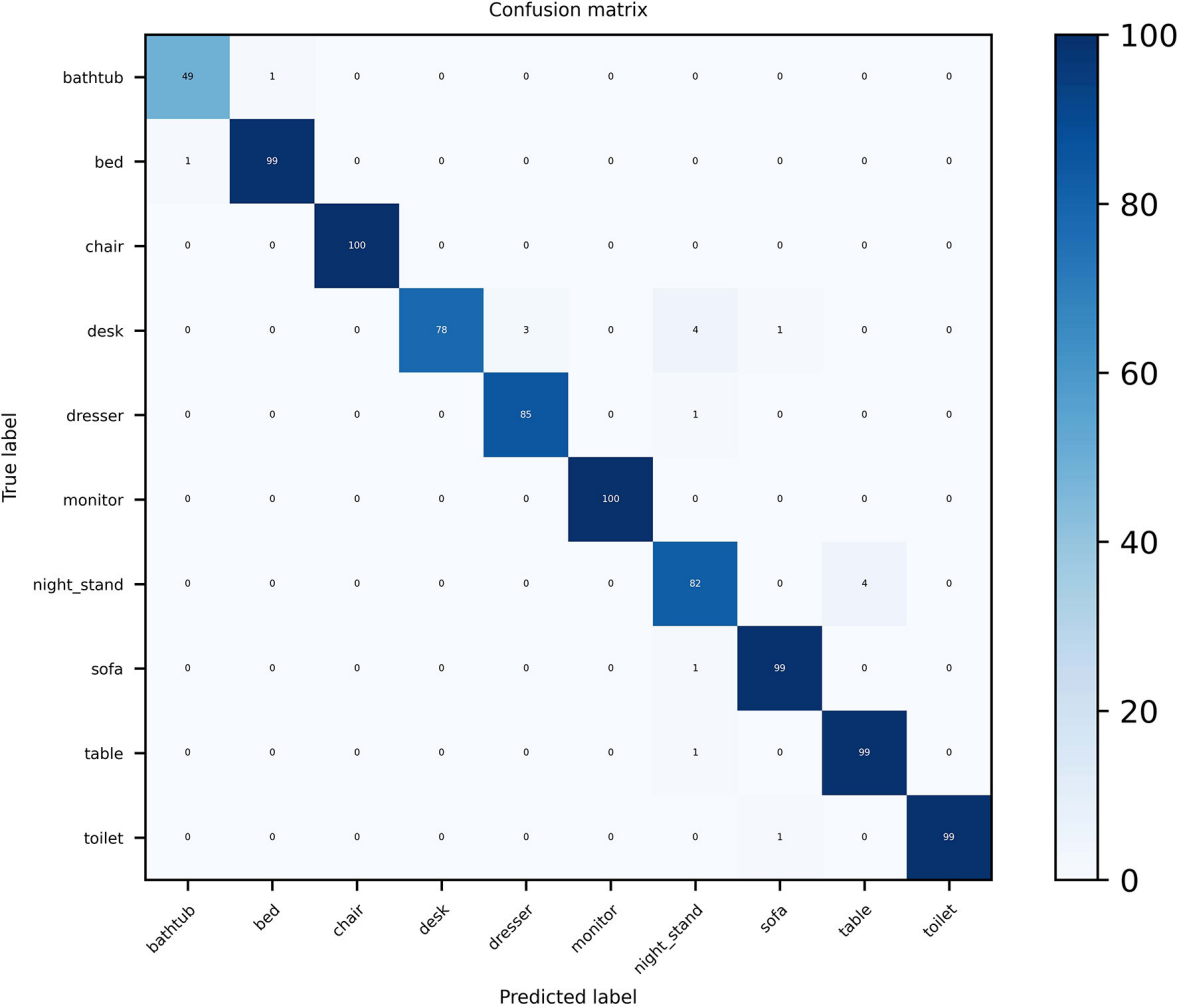
Confusion matrix visualization of MVMSAN on ModelNet10.

For the ModelNet10 dataset, it can be seen from Figure 6 that our MVMSAN also achieved 100% classification accuracy on the chair and monitor categories. In some views, desk, dresser, sofa and other 3D models have high similarity. The existing networks will confuse the feature information of 3D models and cause classification errors. However, our MVMSAN model still has high classification performance for this situation. For example, 78 samples are correctly classified among the 86 the desk models in Figure 6.

The data in the figure is enough to demonstrate the superiority of our approach on the model classification task. Especially for view features with high similarity, our network model is still able to achieve high classification prediction performance.

### 3.7. Comparison with other methods

We compared the classification performance of voxel-based methods [3DShapeNets (Wu et al., 2015), VoxNet (Maturana and Scherer, 2015), and Pointgrid (Le and Duan, 2018)], point cloud-based methods [PointNet (Qi et al., 2017a), PointNet++ (Qi et al., 2017b), MO-Net (Joseph-Rivlin et al., 2019) and DGCNN (Wang Y. et al., 2019) and view-based methods [MVCNN (Su et al., 2015)], GVCNN (Feng et al., 2018), MHBN (Yu et al., 2018), RN (Yang and Wang, 2019), and HMVCN (Liu et al., 2021)]

As shown in Table 6, the proposed MVMSAN outperformed other deep learning methods. Compared with the most classical multi-view-based model classification method (MVCNN), MVMSAN improved OA and AA by 5 and 6%, respectively. Compared with the GVCNN, MHBN, and RN methods, MVMSAN showed considerable improvement. HMVCN is a recently proposed model classification method based on bidirectional LSTM, and its OA reached 94.57%. Our method achieved 2.5% higher OA compared to HMVCN. On the ModelNet10 dataset, the MVMSAN method also achieved the best classification performance (98.57% for OA and 98.42% for AA).

**Table 6:** Classification performance comparison with other methods.

**Network**	**Modality**	**ModelNet40**		**ModelNet10**	
		**OA(%)**	**AA(%)**	**OA(%)**	**AA(%)**
3D ShapeNets	Voxel	84.70	77.30	-	83.54
VoxNet	Voxel	85.90	83.00	-	92.00
Pointgrid	Voxel	92.0	88.90	-	-
PointNet	Point Cloud	89.20	86.20	-	-
PointNet++	Point Cloud	91.90	-	-	-
Mo-Net	Point Cloud	92.40	90.30	-	-
DGCNN	Point Cloud	93.50	90.70	-	-
MVCNN	12-Views	92.10	89.90	-	-
GVCNN	12-Views	92.6	-	-	-
MHBN	6-Views	94.12	92.20	95.00	95.00
	12-Views	93.42	-	-	-
RN	6-Views	94.10	-	-	-
	12-Views	94.30	92.30	95.30	95.10
HMVCM	12-Views	94.57	-	95.7	-
**MVMSAN (Ours)**	3-Views	96.35	94.62	97.80	97.65
	6-Views	96.84	95.65	98.56	**98.50**
	12-Views	96.80	95.31	98.57	98.37
	20-Views	**96.96**	**95.68**	**98.57**	98.42

The bold values represent the best performance.

The excellent performance of our MVMSAN method on the two ModelNet datasets is attributed to three factors: (1) ResNest removes the last fully connected layer and adds an adaptive pooling layer. It can prove that the relationship between view channels can increase the receptive field of view feature extraction, so that the network obtains more detailed features from the input data related to the output. (2) Using the output result of SoftPool as the Query vector of attention can realize the refined down-sampling processing of view feature information, and effectively solve the problem of insufficient extraction and loss of detailed information in the process of view feature extraction. (3) MBConv is employed to process the calculation results of Query and Key of attention. It can enhance the generalization of the model, thereby improving the classification accuracy.

## 4. Conclusion

In this paper, we proposed a multi-view SoftPool attention convolutional network framework, MVMSAN, for 3D model classification. The traditional method does not treat each view equally in the view feature extraction process, and only extracts the feature information that is considered important. This causes the problem of insufficient extraction of the view refinement feature information and loss. Our proposed SoftPool attention convolution framework could achieve refined down-sampling processing for all view features equally, thereby obtaining more useful information from the input data related to the output results, improving the generalization of the model, and achieving high-precision 3D model classification. To better evaluate our network framework, we conducted several experiments to validate the impact of each component of the framework. The experimental results demonstrate that our framework has achieved better classification accuracy on the ModelNet40 and ModelNet10 datasets compared to other advanced methods.

## Data availability statement

Publicly available datasets were analyzed in this study. This data can be found here: http://modelnet.cs.princeton.edu/.

## Author contributions

WW and XW brought up the core concept and architecture of this manuscript. XW wrote the paper. GC and HZ corrected the sections of the manuscript. All authors contributed to manuscript revision, read, and approved the submitted version.

## Funding

This study was supported by the Natural Science Foundation of Shanghai under Grant No. 19ZR1435900.

## Conflict of interest

The authors declare that the research was conducted in the absence of any commercial or financial relationships that could be construed as a potential conflict of interest.

## Publisher's note

All claims expressed in this article are solely those of the authors and do not necessarily represent those of their affiliated organizations, or those of the publisher, the editors and the reviewers. Any product that may be evaluated in this article, or claim that may be made by its manufacturer, is not guaranteed or endorsed by the publisher.
